# *Bacillus subtilis* and *Bacillus amyloliquefaciens* Mix Suppresses Rhizoctonia Disease and Improves Rhizosphere Microbiome, Growth and Yield of Potato (*Solanum tuberosum* L.)

**DOI:** 10.3390/jof9121142

**Published:** 2023-11-25

**Authors:** Vladislava S. Maslennikova, Vera P. Tsvetkova, Evgenia V. Shelikhova, Marina P. Selyuk, Tatyana Y. Alikina, Marsel R. Kabilov, Ivan M. Dubovskiy

**Affiliations:** 1 Laboratory of Biological Plant Protection and Biotechnology, Novosibirsk State Agrarian University, Dobrolubova Str. 160, 630039 Novosibirsk, Russia; vladislava.maslennikova@mail.ru (V.S.M.);; 2 Laboratory of Biotechnology of Microorganisms and Plants, Tomsk State University, 634050 Tomsk, Russia; 3 Institute of Chemical Biology and Fundamental Medicine, Siberian Branch of the Russian Academy of Sciences, 630090 Novosibirsk, Russia

**Keywords:** biocontrol, stem canker, plant physiology, black scurf, diseases

## Abstract

Black scurf and stem canker caused by *Rhizoctonia solani* is a significant disease problem of potatoes. Currently, chemical methods are the primary means of controlling this pathogen. This study sought to explore an alternative approach by harnessing the biocontrol potential of a bacterial mix of *Bacillus subtilis* and *Bacillus amyloliquefaciens* against black scurf, and to determine their effect on rhizosphere microorganisms of soil microbiota. This study showed that these bacteria demonstrate antagonistic activity against *Rhizoctonia solani*. Reduced damage to potato plants during the growing season in Siberia was observed. The index of disease development decreased from 40.9% to 12.0%. The treatment of tubers with this mix of bacteria also led to a change in the composition of the rhizosphere microbiota (according to CFU, 16S and ITS sequencing). This effect was accompanied by a positive change in plant physiological parameters (spectrophotometric analysis). The concentration of chlorophyll in potatoes with the bacterial mix treatment increased by 1.3 fold (*p* ≤ 0.001), and of carotenoids by 1.2 fold (*p* ≤ 0.01) compared with the control. After bacterial mix treatment, the length of the aerial parts of plants was 1.3 fold higher (*p* ≤ 0.001), and the number of stems 1.4 fold higher (*p* ≤ 0.05). The yield of potatoes was increased by 8.2 t/ha, while the large tuber fraction was increased by 16% (*p* ≤ 0.05). The bacteria mix of *Bacillus subtilis* and *Bacillus amyloliquefaciens* suppressed the plant pathogenic fungus *Rhizoctonia solani*, and simultaneously enhanced the physiological parameters of potato plants. This treatment can be used to enhance the yield/quality of potato tubers under field conditions.

## 1. Introduction

Potato (*Solanum tuberosum* L.) is a globally important food crop, and black scab is a major and widespread tuber disease problem for potato production. It is caused by *Rhizoctonia solani* Kühn (teleomorph *Thanatephorus cucumeris* (A.B. Frank) Donk) [1]. The presence of the fungus *R. solani* leads to worldwide crop losses of 25–30% every year, rising to 60–80% for highly susceptible varieties [2,3].

Although infection from tubers is a factor in disease dynamics and a target for control agents, soil as an inoculum source is also important. Severe infection levels can occur from infected soil even when using disease-free seed tubers [1]. This increases the significance of monitoring and controlling not only tuberous, but also soil populations of *R. solani* [4].

Currently, chemical fungicides are used against potato rhizoctonia disease. Their main advantages are their efficiency, mass availability and high activity [5]. The main drawbacks are the dangerous toxic effect to humans and animals, as well as the death of beneficial insect pollinators (e.g., bees and bumblebees). A growing awareness of the dangers associated with pesticides has underlined the need for developing alternative, biological methods to control plant diseases [5].

The natural phenomenon of antagonism of microorganisms in relation to phytopathogens is the basis for the microbiological approach to protect plants from disease. This makes it a promising methodology in terms of its efficiency and environmental friendliness [6,7].

Biological control based on beneficial bacteria is an effective solution for the suppression of diseases transmitted through the soil [8]. Some bacteria alter plant metabolism to increase disease resistance. This is expressed in morphometric indicators, and in a change in the biochemical processes of oxidation. The pathogen also causes significant changes in the plant metabolism, and there usually are several distinct stages in the development of a plant response to infection [9]. Many proteins are involved in all these stages, including enzymes, and peroxidase plays an important role [10]. This enzyme is considered as one of the most important biochemical factors for plant protection from pathogenic organisms. Peroxidase is associated with elicitor receptors in the first line of cell defense against the pathogen [11]. It participates both in the formation of a mechanical barrier to the infection [12] and in the generation of reactive oxygen species fatal to the pathogen.

Aerobic spore-forming bacteria of the genus *Bacillus* are widespread in the biosphere. They are heterotrophic saprophytes occupying various ecological niches in soil and aquatic ecosystems. However, in terms of their total biomass, they are most prevalent in soils [13]. Representatives of the genus *Bacillus* can populate the rhizosphere of plants using nutrients produced by plants in the form of root exometabolites. They are also able to synthesize biocontrol substances, phytohormones and vitamins that act to support plants [14,15,16]. Bacilli are involved in many different relationships with plants: they can be causative agents of diseases [17,18], promote sustainable plant growth and development [19] or protect the plant from infections [20]. In soil, bacteria of the genus *Bacillus* are found in the form of spores or vegetative cells. At soil temperatures close to 0 °C, most bacilli form spores. In this state, microorganisms are highly resistant to external factors. *Bacillus subtilis* strains have multiple effects on pathogens: they produce antibiotics, are antagonists against phytopathogens and increase plant immunity. In addition, in most cases they exhibit a stimulating effect on plant growth [21]. Most of the identified antibiotics produced by bacteria are peptides. However, in recent years, an increasing number of reports indicate that representatives of this group of microorganisms also produce antibiotic substances belonging to other classes of compounds [22]. In addition, industrial strains of bacteria produce a wide range of enzymes (amylolytic, cellulolytic, proteolytic). This promotes the activation of humification processes in the soil and, as a consequence, increases its fertility [23].

Toxic effects of pathogens on plants are known to manifest as the development of oxidative stress and the formation of reactive oxygen species (ROS). ROS can initiate lipid peroxidation, resulting in damage to membrane structures. In addition, the lipid peroxidation products (4-hydroxyalkenals, malondialdehyde, etc.) possess mutagenic activity and block cell division [24].

This development of oxidative stress in plants can be studied using malondialdehyde (MDA) as a biological indicator [25], and chlorophyll fluorescence kinetics can be used as an informative tool for studying of the effects of different environmental stresses on photosynthesis [26]. The chemical composition of potatoes determines processing quality and is influenced by several other factors, including production area, cultivars, soil and climate, agricultural practice, storage and commercialization conditions [27].

Complex and diverse plant-bacterial interactions take place in the potato rhizosphere [28]. Rhizosphere-inhabiting bacteria include free-living rhizospheric bacteria (e.g., *Pseudomonas* spp., *Bacillus* spp., *Streptomyces* spp., *Burkholderia* spp., *Azospirillum* spp., etc.) and bacteria that form specific symbiotic relationships with plants: *Rhizobium* spp. and *Frankia* spp. [29]. These improve the nitrogen and phosphorus nutritional status of plants, help strengthen their immune system and increase yield by 20–30% or more [30,31].

The activity of introduced microorganisms depends on their survival in the rhizosphere [32]. Their concentration in the rhizosphere and density of root colonization depends on the correct selection of strains, and the use of agronomically useful mixtures and associations [33,34]. Among the aerobic spore-forming bacteria, *Bacillus subtilis* has the greatest importance as a basis for biological preparations against plant diseases. It is the largest source of *Bacillus* derived antibiotics that inhibit the growth of phytopathogenic microorganisms. Some of these are already widely used in medicine, veterinary medicine, agriculture, food industry, etc., including the antibiotics: bacilisin, subtilin, bacillin, bacillomycin, iturin, etc. [35]. Over the past 20 years, a number of researchers [36,37,38,39] have investigated *Bacillus* strains to create effective biological products and plant protection products. Such preparations are promising, since the strains included in their composition produce biologically active substances, hormones and vitamins. This allows them to adapt to various soil conditions and is important when used in integrated plant protection [12]. Successful control of *R. solani* with bacteria of the genus *Bacillus* on various cultures has been previously reported [40,41]. However, the specific effects of *Bacillus* on *R. solani*, as well as on the bacterial and fungal soil microbiota, remain undetermined. In addition, the physiological and biochemical responses of the potato plant have not been resolved.

The aim of this research is therefore to assess the effect of a mix of *B. subtilis* and *B. amyloliquefaciens* on the soil microbiota and on the physiological parameters of potatoes.

## 2. Results

### 2.1. Growth Promotion Effects over the Growing Season

**Figure 1:**
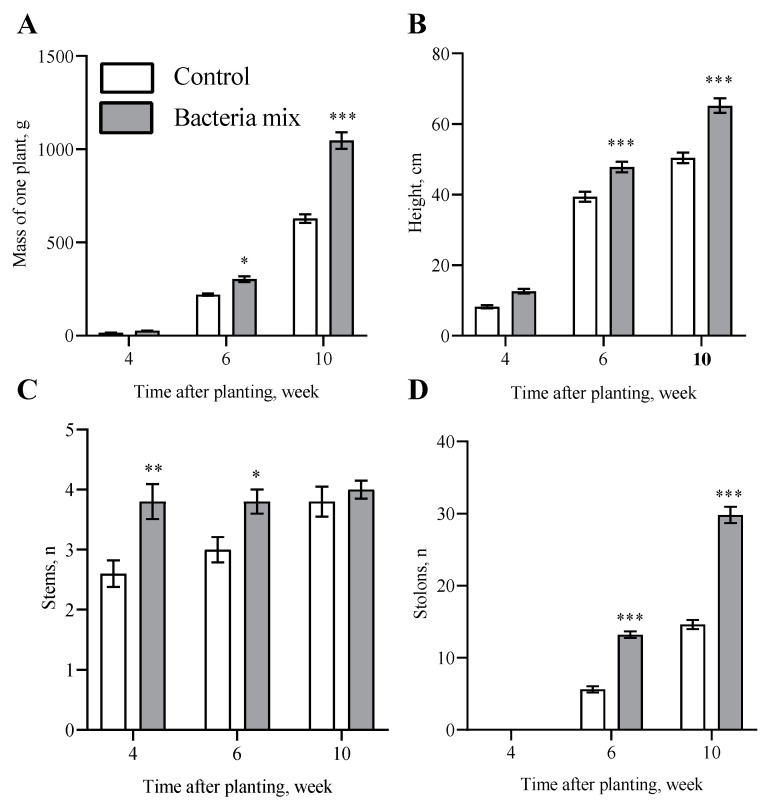
Effect on potato growth at 4, 6 and 10 weeks post inoculation of tubers: Per plant values for (**A**) mass, (**B**) height, (**C**) stem number, (**D**) stolon number. Treatment: bacteria mix (*B. subtilis* and *B. amyloliquefaciens* 1 × 10^6^ CFU/mL), Control: water (* *p* ≤ 0.05, ** *p* ≤ 0.01, *** *p* ≤ 0.001 compared with the control at the same time point).

The weight per plant had increased on the 6th week after planting by 1.3 fold (*p* = 0.027) and on the 10th week by 1.6 fold (*p* < 0.0001) under the influence of bacteria mix treatment compared with the control (Figure 1A). The length of the aerial parts of plants was 1.2 fold (*p* = 0.0002) and 1.3 fold (*p* < 0.0001) higher on 6th and 10th week, respectively, post bacteria mix treatment compared with the control (Figure 1B and Figure S1). Stems in the bacteria mix treatment started to emerge earlier than in controls: 3.8 vs. 2.6 on 4th week (*p* = 0.0012) and 3 vs. 3.8 on 6th week (*p* = 0.043) post planting (Figure 1C). The number of stolons was higher: 2.3 fold (*p* < 0.0001) on 6th week and 2 fold (*p* < 0.0001) on 10th week in the post bacteria mix treated plants compared with control treated plants (Figure 1D).

### 2.2. Inhibition of Rhizoctonia solani, In Vitro and over the Growing Season

The results demonstrate inhibition of *R. solani* by the *B. amyloliquefaciens* and *B. subtilis* mix both in vitro and in the field, where disease progression was suppressed.

**Figure 2:**
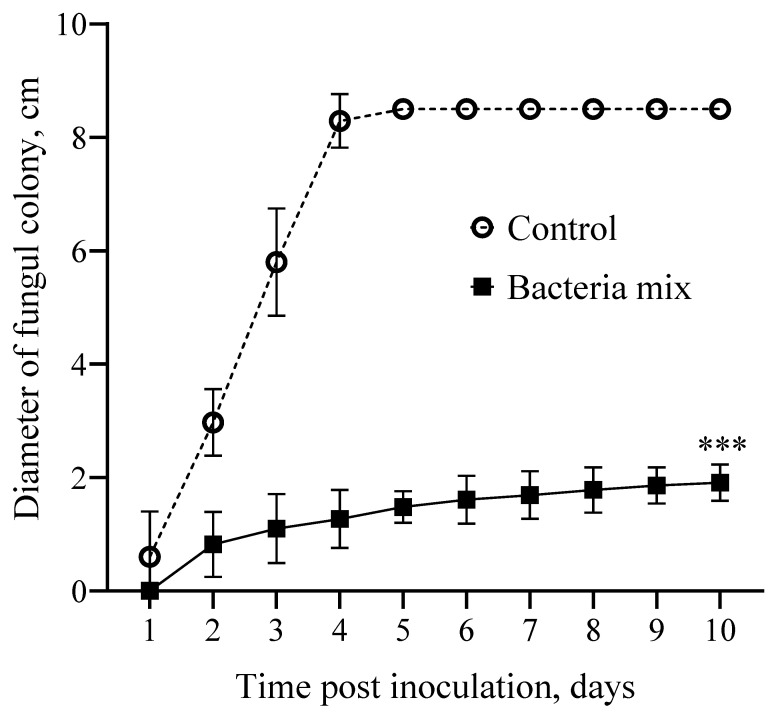
Effect on growth of *Rhizoctonia solani* in vitro. Treatment: bacteria mix (*B. subtilis* and *B. amyloliquefaciens* 1 × 10^6^ CFU/mL), Control: water (*** *p* ≤ 0.001 compared with the control from second to tenth day post inoculation).

The diameter of the Rhizoctonia colony was 5.2 fold smaller on the 3rd day post bacteria mix treatment compared with the control. The inhibitory activity (IA) of bacteria mix treatment on the fungus was 81.0%. For all time points of the experiment this trend continued, and the average IA was 81.7% (df = 1; F(1, 180) = 7870; *p* < 0.0001) (Figure 2 and  Figure S2).

The number of stems of plants affected by *R. solani* post bacteria mix treatment decreased by 4-fold and there were no stems with 3 or 4 points of damage on the 4th week after planting (Table 1). The number of stolons increased by 2–2.3 fold for the plants with bacteria mix treatment compared with the control, when no differences due to *R. solani* were detected (Figure 1D, Table S1). The biological effectiveness of bacteria mix treatment at 4 weeks was 100%, at 6 weeks—86.8%, and at 10 weeks—70.7% (Table 1).

**Figure 3:**
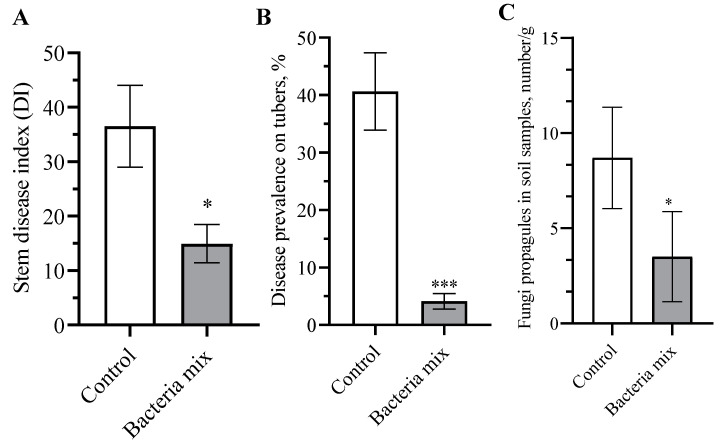
Effect on development of *Rhizoctonia solani* in the field (**A**) stem disease index, (**B**) tuber disease prevalence, (**C**) soil prevalence. Treatment: bacteria mix (*B. subtilis* and *B. amyloliquefaciens* 1 × 10^6^ CFU/mL), Control: water, measured on the 10th week post planting (* *p* ≤ 0.05; *** *p* ≤ 0.001).

The index of the development of the disease on stems in plants with bacteria mix treatment decreased from 40.9% to 12.0% (t = 2.604, df = 18; *p* = 0.017) compared with controls on the 10th week after planting (Figure 3A). The treatment of tubers with these bacterial strains significantly improved the quality of the new crop compared to the control (Figure S3). The prevalence of *Rhizoctonia* in the bacteria mix treatment decreased to 4.1% compared with the control (40.67%) (t = 5.321; df = 18; *p* < 0.0001) (Figure 3B). We evaluated the effect of bacteria mix on *R. solani* in the rhizosphere of potato. The results showed a decrease in the number of *R. solani* propagules in soil samples in the treatment compared with the controls of 4.2 fold (*p* = 0.015, df = 18) (Figure 3C).

### 2.3. Effect on Plant Physiological Parameters over the Growing Season

**Figure 4:**
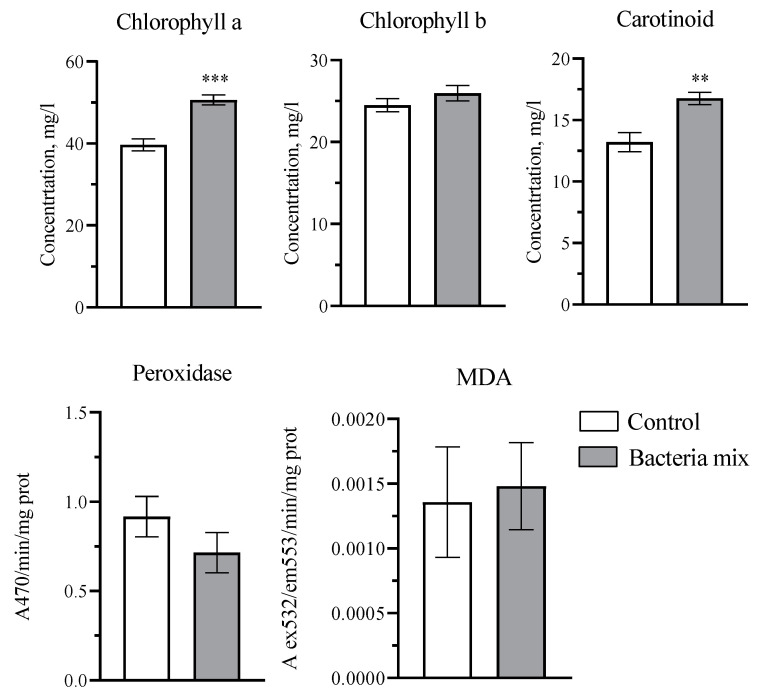
Effect on plant physiological parameters: photosynthetic pigments, peroxidase activity and malondialdehyde (MDA). Treatment: bacteria mix (*B. subtilis* and *B. amyloliquefaciens* 1 × 10^6^ CFU/mL). Concentrations measured in potato leaves on the 4th week post planting (** *p* ≤ 0.01, *** *p* ≤ 0.001 compared with the control).

The concentration of chlorophyll a in potatoes with bacterial mix treatment increased by 1.3-fold (*p* < 0.0001; t = 5.789; df = 18), and of carotenoids by 1.2 fold (*p* = 0.0012; t = 3.848; df = 18) compared to the control treatment on the 4th week post planting. The chlorophyll b concentration, activity of peroxidase and the concentration of malondialdehyde in potato leaves was not changed significantly (Figure 4).

### 2.4. Effect on Soil Bacterial and Fungal Communities

**Figure 5:**
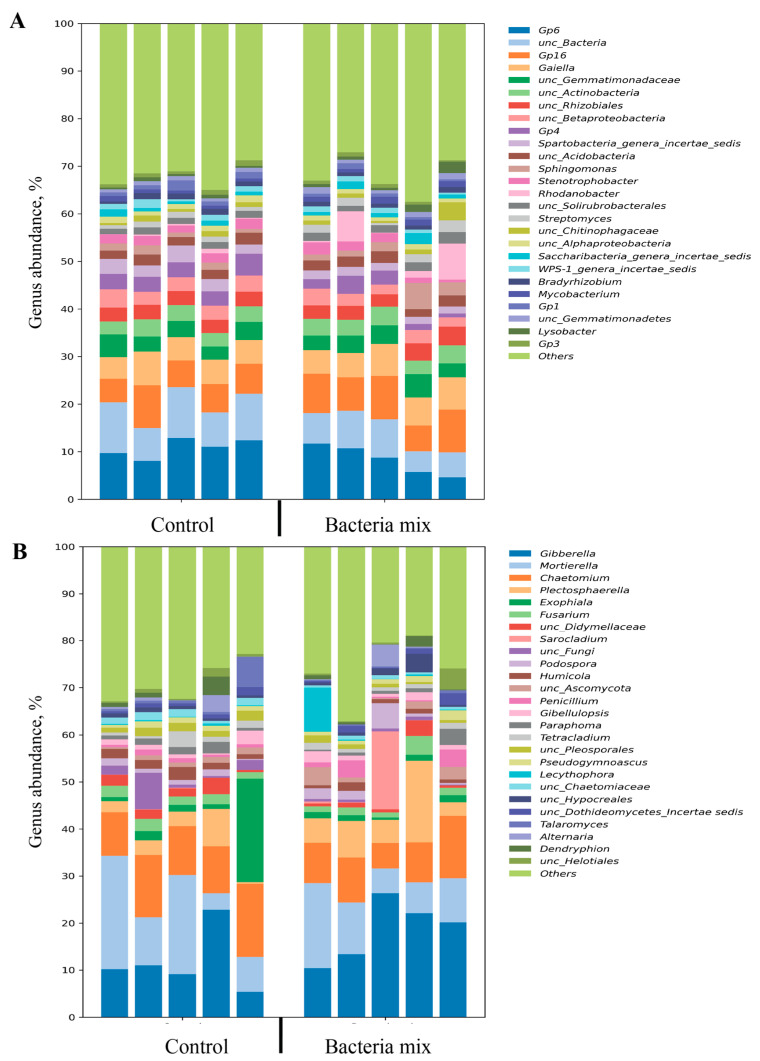
Relative abundance of genera in metagenomic analysis of soil planted with potatoes (**A**) bacterial (16S) (**B**) fungal (ITS). Treatment of tubers: bacteria mix (*B. subtilis* and *B. amyloliquefaciens*, 1 × 10^6^ CFU/mL), Control: water, measured on the 4th week post planting.

The major bacteria (more than 10% relative abundance) in the soil for both bacteria mix and control treatments were classified with 16S and belonged to 3 phyla: *Acidobacteria*, *Proteobacteria* and *Actinobacteria*. Altogether 357 genera of bacteria were detected, and the most prevalent (more than 3% relative abundance) were classified as Gp6 (*Bacteroidaceae*), Gp16, *Gaiella*, *Actinobacteria*, *Rhizobiales*, *Gemmatimonas*, *Betaproteobacteria*, Gp4, *Spartobacteria* genera incertae sedis, *Sphingomonas* or *Rhodanobacter* (Figure 5A).

Sequence analysis did not show any significant differences in abundance of the main genera of bacteria (more than 1% relative abundance) between bacteria mix and control treatments, except for *Spartobacteria* which declined (*p* = 0.01) with bacteria mix treatment compared with the control (Figure 5A).

The bacteria mix treatment induced the increase in abundance of some minor genera of bacteria in the soil, i.e., *Hyphomicrobiaceae, Aeromicrobium, Pedobacter, Microbacterium, Rhizobium, Phyllobacteriaceae* compared with the control (*p* < 0.05). Bacteria from the genera Subdivision3 genera incertae sedis, *Rhodospirillaceae*, *Latescibacteria* genera incertae sedis, *Massilia*, Gp5, Gp25, Gp2 and *Terrimonas* showed a decline in abundance in the bacteria mix treatment compared with the control (*p* < 0.05) (Figure 5A).

The major fungi (more than 10% relative abundance) in the soil for bacteria mix and control treatments were classified with ITS to the phyla *Ascomycota, Mortierellomycota, Basidiomycota* and some were unclassified. In total, 158 genera of fungi were detected. The most prevalent (more than 3% relative abundance) were *Sordariomycetes, Ascomycota, Hypocreales, Mortierellaceae, Mortierella* and *Fusarium* (Figure 5B).

The abundance of the fungal genus *Hypocreales* was higher, and that of the unclassified fungi was lower with bacteria mix treatment of potato tubers compared with the control (*p* < 0.05) (Figure 5B).

The bacteria mix treatment also resulted in a decrease in abundance of minor genera of fungi *Chaetomiaceae* and *Hypocreaceae* in the soil compared with the control (*p* < 0.05) (Figure 5B).

**Figure 6:**
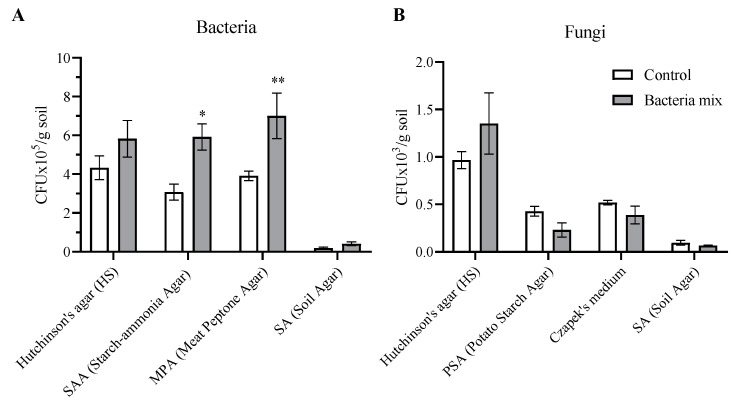
Counts of bacteria (**A**) and fungi (**B**) on different media from dry soil planted with potatoes. Isolation on SAA and PSA media for mineral nitrogen assimilators, on MPA media for ammonifiers, and on HS media for cellulolytic activity. Treatment of tubers: bacteria mix (*B. subtilis* and *B. amyloliquefaciens*, 1 × 10^6^ CFU/mL), Control: water, measured on the 4th week post planting (* *p* ≤ 0.05; ** *p* ≤ 0.01 compared with controls).

We evaluated the number of colony forming units (CFUs) from the soil from the rhizosphere of the plants on various media. The total number of bacteria at the 4th week after bacterial treatment was increased by 2 fold on media specific for bacteria assimilating mineral nitrogen (PSA) (*p* = 0.01) and media specific for bacteria utilizing organic forms of nitrogen (MPA) (*p* = 0.007) compared with controls (Figure 6A).

Fungi cultivated on HS media with the ability to degrade cellulose were highly represented in the soil compared with those cultivated on other artificial media types (PSA, Czapek, SA). The overall number of fungi did not change with bacteria mix treatment (Figure 6B). However, fungi from the genus Fusarium decreased with bacterial treatment compared with controls (t = 2.684; df = 8; *p* = 0.02) (PDA medium) (Figure S4B). Bacteria mix treatment did not have any effect on actinomycetes abundance tested with microscopic colony determination on Hutchinson’s medium, starch-ammonia agar (SAA) and Soil Agar (Figure S4A). Thus, a positive effect of the bacteria mix treatment, increasing nitrogen-fixing bacteria was noted, and, at the same time, no negative effect on the fungal saprotrophic microflora of the soil involved in the decomposition of organic matter was observed.

### 2.5. Effect on Biological Productivity and Quality of Tubers

**Figure 7:**
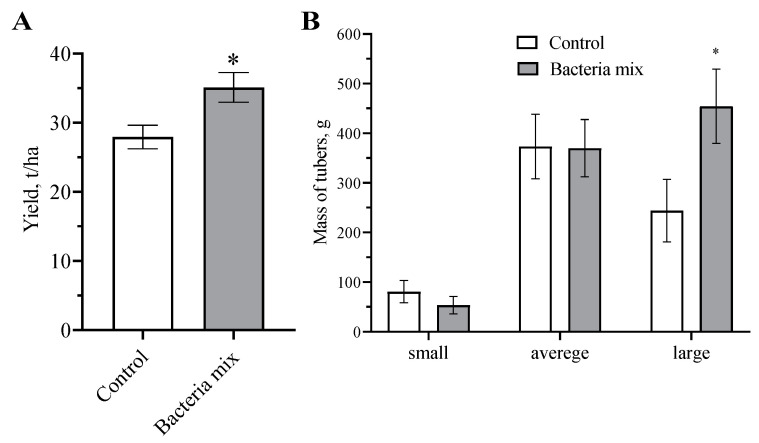
Effect on harvest of Potato (*Solanum tuberosum*) (**A**) yield per hectare, (**B**) distribution of tuber size fraction. Treatment of tubers: bacteria mix (*B. subtilis* and *B. amyloliquefaciens*, 1 × 10^6^ CFU/mL), Control: water. (* *p* ≤ 0.05 compared with the control).

These findings showed that the bacterial mix treatment had a positive effect on the yield of potato tubers (Figure 7 and Figure S1). Tubers were predominantly larger (1.8 fold higher) with bacteria mix treatments compared with controls (*p* = 0.035; t = 2.72; df = 24) (Figure 7). A yield increase on 8.2 t/ha in the bacteria mix (*B. subtilis* and *B. amyloliquefaciens*) treatment compared with control treatment was detected after harvesting (*p* = 0.0311; t = 2.610; df = 8) (Figure 7).

We also analyzed 11 indicators of the biochemical composition of tubers: moisture, dry matter, crude fiber, fiber in dry matter, crude ash, ash on dry matter, crude protein, protein on dry matter, total acidity, pH and nitrates. All of these parameters in harvested tubers were not changed post bacteria mix treatment compared with control treatment (Figures S5 and S6).

## 3. Discussion

We found that preplant treatment of potato tubers with the bacteria mix of *B. amyloliquefaciens* and *B. subtilis* had a plant growth-promoting effect, and a fungicidal effect on plant pathogenic fungi. The bacteria mix treatment affected soil microbiota, the biochemistry of the plant during the growing season and the quality of new generation tubers. The bacteria mix suppressed *Rhizoctonia solani* not only on the stems and tubers, but also in the soil. It enhanced the processes of photosynthesis in plants by increasing the level of chlorophyll a and of carotenoids. Overall, it led to an increase in yield and an improvement in the quality of tubers.

Some studies confirm that *Bacillus* can play a role in promoting plant growth [42]. *Bacillus* strains promote plant growth directly or indirectly [43]. For example, *B. licheniformis* MH48 significantly increased the growth of seedlings of *Camellia oleifera*, particularly in root dry weight, by 7.42 g plant^−1^, which was 1.7-fold greater than that of the control [44]. In this study, during the pre-planting treatment of tubers, the growth-promoting effect of the bacterial *B. amyloliquefaciens* and *B. subtilis* strains was shown as an increase in the values of morphometric parameters of plants during the growing season, such as: mass per plant, number of stems and stolons, and length of the aerial parts. We were able to show that concentrations of both chlorophyll a and carotenoids increased, which can positively affect the productivity of potatoes.

One of the most important factors in plant productivity is the rate of photosynthesis, which depends on environmental conditions, air and mineral nutrition [45,46]. These conditions determine the content of chlorophyll in the leaves and the functioning of the entire photosynthetic apparatus. An increase in photosynthesis then results in an increase in carbohydrate accumulated during the growing season. Potato tubers contain a high percentage of starch, a major storage carbohydrate, so this can directly determine the mass of marketable plant products produced [47,48]. In previous studies, a correlation was shown between chlorophyll content and yield [49], and variability of chlorophyll content in plants with different productivity [50].

We found that the bacteria mix of *B. amyloliquefaciens* and *B. subtilis* strongly suppresses phytopathogenic fungus *Rhizoctonia solani* growth in vitro and on stems, tubers and in the rhizosphere of the potato plants. The suppression of *Rhizoctonia* by specific microorganisms is often associated with the formation of secondary metabolites that are toxic to the pathogen [49,51]. The compounds used for biocontrol of *R. solani* are usually antibiotics or enzymes that lyse the cell walls of the fungi [50,52]. It has been demonstrated that the ability of many strains of rhizobacteria to suppress plant pathogens depends on their ability to produce secondary metabolites that inhibit pathogens. Such metabolites include antibiotics, siderophores, bacteriocins and cyanide [6]. The strains used in this study appear to release antifungal substances in the rhizosphere of potatoes, resulting in a significant reduction in the damage to the stems and tubers from rhizoctoniosis. There are several examples of microorganisms that stimulate growth of plants while suppressing *Rhizoctonia solani*. For example, yeast colonies on sugar beet roots have been shown to stimulate growth and reduce the prevalence of *Rhizoctonia* [53]. Many strains of mycorrhizal fungi that promote the development of rhizobacteria also inhibit *Rhizoctonia* and have been shown to promote plant growth and reduce both the development of the disease on the stems and the black scab of potato tubers in field trials [54,55].

In this study, analysis of sequence data showed that with preplant treatment of potato tubers with the mix of *B. amyloliquefaciens* and *B. subtilis*, the amounts of bacteria of the *Hyphomicrobiaceae*, *Aeromicrobium*, *Pedobacter*, *Microbacterium*, *Rhizobium*, *Phyllobacteriaceae* were significantly increased. A group of bacteria of the order of *Rhizobiales*, capable of binding inorganic atmospheric nitrogen and producing organic nitrogen-containing substances [56], also increased their abundance in the soil, which favorably affected the growth and development of potatoes. The presence of *Fusarium* fungi and the abundance of some other fungi identified by ITS analysis of the soil (*Chaetomiaceae* and *Hypocreaceae*) was decreased post bacteria (*B. amyloliquefaciens* and *B. subtilis)* mix treatment. At the same time, there was an increase in the number of some bacteria that assimilate organic and mineral nitrogen and play an important role in the fixation of atmospheric nitrogen and the supply of accessible forms of plant nitrogen [57], as confirmed by the sequence data. However, the bacteria mix of *B. amyloliquefaciens* and *B. subtilis* did not affect cellulose-degrading actinomycetes, which are an important link in the natural decomposition process, which ensures the return of carbon fixed during photosynthesis into the atmosphere in the form of CO_2_ [58]. The global role of microorganisms in this process is determined by the fact that neither animals nor plants are able to decompose cellulose, which is one of the main components of plant residues (its content is from 15 to 60% of the mass of plants) and its degradation is an important contributor to soil processes and properties.

## 4. Conclusions

The bacteria mix of *Bacillus amyloliquefaciens* and *B. subtilis* had an antagonistic activity against *Rhizoctonia solani* on potatoes and reduced the damage to plants during the growing season. The treatment of tubers with this bacteria mix led to a change in the composition of the soil microbiota: the number of bacteria assimilating mineral nitrogen and organic nitrogen (ammonifiers) increased significantly. In addition, the soil population of the fungus *Rhizoctonia solani* decreased under the influence of the bacteria mix. This effect was accompanied by an improvement in the morphometric and physiological parameters (photosynthetic pigments) of the plants and an increase in the yield of tubers. To reduce the prevalence of the phytopathogenic fungus *Rhizoctonia solani* on stems, stolons and potato tubers, to stimulate the rhizoversal microbiota and increase the productivity and quality of the new crop, a preplant treatment of potato tubers with this bacteria mix at a concentration of 1 × 10^6^ CFU/mL is recommended.

## 5. Materials and Methods

### 5.1. Experimental Design

The studies were conducted from 2017 in the fields of the agricultural complex “Gardens of the Giant”, located in the village of Koltsovo (54°56′20″ N 83°11′00″ E), Novosibirsk region. The experimental site had a plot size of 100 hectares. The soil of the experimental plot was a medium alfisol with a humus content in the 0–30 cm layer—3.35–4.14%, easily hydrolyzed nitrogen—2.01–2.36 mg/100 g of soil, mobile phosphorus—15.0–19.3 mg/100 g of soil, exchange potassium—7.99–10.3 mg/100 g of soil, pH of salt extract—6.1–6.5.

The bacterial strains *Bacillus amyloliquefaciens* VKPM B-10642, *B. amyloliquefaciens* VKPM B-10643 and *B. subtilis* VKPM B-1064 were provided by Scientific Production Company Issledovatelskiy Tsentr, 630559, Novosibirsk Region, Koltsovo Science City, Industrial Zone, Building 200 and were verified by 16s sequencing and NCBI blast analysis (Table S2). The bacteria mix consists of spore biomass and excipients, i.e., the nutrient medium after culturing the strains, saturated with growing cells (1 × 10^9^ Per ml of each strain).

The potatoes were an early ripe variety of Rosara potatoes provided by Solana-Agro Service LLC (Samara, Russia). The tubers had an initial prevalence of rhizoctonia of 16.7%.

The preplant treatment was carried out with a suspension 1 × 10^6^ CFU/mL of each bacteria *Bacillus amyloliquefaciens* VKPM B-10642, *B. amyloliquefaciens* VKPM B-10643, *B. subtilis* VKPM B-10641, by soaking tubers for 60 min (10 L of suspension per 1 ton of tubers). The control tubers were treated with water.

The experimental design is detailed in supplementary Figure S7. At the 4th, 6th and 10th week after planting, 10 potato plants were selected from each replicate (*n* = 50). The height, the number of stems and stolons, the plant weight and the prevalence of rhizoctoniosis were assessed. On the 4th week after planting, leaves were selected for biochemical studies (*n* = 20) and soil samples taken for microbiological analysis (*n* = 20). After harvesting potatoes, the yield and quality of tubers were evaluated (*n* = 50).

### 5.2. Bacterial Antifungal Activity against R. solani (In Vitro)

*Rhizoctonia solani* was isolated from rhizoctoniosis-affected potato tubers of the Rosara variety (Russia, Novosibirsk) in 2016. The ITS gene of the fungus was sequenced and verified by NCBI blast analysis (Table S2).

Screening experiments were performed in vitro using an agar block technique. A suspension of the bacteria of 10^6^ CFU/mL was introduced into the nutrient medium (potato-glucose agar) at a temperature of 36–37 °C. The inoculated medium was poured into Petri dishes. Then a block with diameter of 10 mm, cut from a *R. solani* colony, was placed in the center. Plates were incubated at a temperature of 22 °C and measurements taken daily for 10 days. Tenfold replicates were used, comparing the diameter of the fungal colonies in treated samples with media-only controls to determine inhibitory activity.

### 5.3. Morphometric and Disease Analysis of Plant Material

The following morphometric indicators were measured: length and number of stems, mass per plant, total number of stolons and number of damaged and fallen stolons.

Measurement of the incidence of rhizoctoniosis on sprouts and stems and of morphometric indicators was carried out 4, 6 and 10 weeks after planting. The percentage of damaged and fallen stolons was calculated. Disease severity was expressed following a rating scale based on the percentage of lesions on the stem [59]: 0 (no lesion), 1 (more than 1% less than 5%), 2 (more than 5% less than 25%), 3 (more than 25% less than 50%), 4 (more than 50% less than 75%) and 5 (more than 75%). A *Rhizoctonia solani* disease development index, DI, was calculated for stems using the formula DI = Σ [0 (n0) + 0.2 (n1) + 0.4 (n2) + 0.6 (n3) +0.8 (n4) +1 (n5)] × 100/(N total), where n = number of corresponding grade plants evaluated and N = total number of stems or stolons.

A sample of 200 tubers was taken from at least ten places from each treatment. The tubers were washed, carefully inspected, and divided into healthy tubers and those affected by *Rhizoctonia*. Manifestations of rhizoctonia disease such as net necrosis, deep spotting and the presence of sclerotia (single, or occupying 1/10, 1/4 or 1/2 of the tuber surface) were measured to indicate the origin and degree of damage to the tuber surface. Seed potato standards include the following sowing quality requirements: damage to the surface of tubers by *Rhizoctonia* from 1/8 to 1/4 of the total tuber surface area in classes is permitted 0.5–3.0%, and damage over 1/4 is not permitted.

### 5.4. Plant Physiology Assay

The content of photosynthetic pigments was determined by spectrophotometry based on the ability of pigments to absorb at specific wavelengths. Extracts of pigments from potato leaves in 70% alcohol were prepared. Carotenoids were determined by spectroscopy at λ = 440 µm, chlorophyll a at λ = 665 µm, chlorophyll b at λ = 649 µm. The measurement of each pigment extract was carried out with ten replicates. The concentrations of chlorophyll a and b in the extract were calculated using the Vernon formula [60]. The concentration of malondialdehyde and peroxidase in the leaves was measured according to a standard methodology used for plant biochemical analysis [61].

For the tuber biochemical parameter assay, the samples for qualitative examinations (8–10 kg of potato tubers) were collected from four field replications for each plot using standard methods. Washed and peeled potato tubers were fragmented, and the following indices were determined with respect to the potato pulp: moisture, ash, total protein, total acidity, pH and fibers [62].

### 5.5. Counts of Bacteria and Fungi in Soil

Five soil samples were taken from the root zone of potatoes, each weighing 100–150 g. A 10 g sample of dry soil was prepared and placed in a flask with sterile water (90 mL). Samples were shaken well and processed via serial dilution. The soil suspensions of the required dilutions were inoculated onto selective media that had been pre-sterilized by autoclaving for 30 min at 1 atm at a temperature of 121 °C.

The total number of bacteria utilizing organic forms of nitrogen was assessed on meat peptone agar (MPA); for bacteria utilizing mineral nitrogen and actinomycetes, starch-ammonia agar (SAA) was used, and Hutchinson’s agar (GN) was used for cellulolytic microorganisms. For assessment of fungi, streptomycin (0.2 g/L) was added to the Czapek’s medium, PSA (Potato Starch Agar) and GN (Hutchinson’s agar) then cooled to 45 °C before pouring into Petri dishes [63].

Each soil sample was inoculated onto 5 artificial nutrient media with 5 replicates. Petri dishes were incubated at 25–27 °C for 7–10 days. The number of colonies of microorganisms (CFUs) in each sample was counted and recalculated per 1 g of air-dried soil. Fusarium was detected on PSA media using microscopic assay [64].

Quantitative determination of the population density of *R. solani* in soil was carried out in the laboratory of biological plant protection and biotechnology of Novosibirsk State Agrarian University using a multiple soil tablets method [65].

### 5.6. 16S and ITS Metabarcoding Sequencing

The soil microbiome was analyzed using 16S rDNA and ITS metagenomic sequencing. Soil samples were collected from the root zone of potatoes, each weighing 100–150 g and frozen at −20 °C. DNA was isolated from 250 mg soil using the DNeasy PowerSoil 462 Kit (Qiagen, Hilden, Germany), with homogenization using TissueLyser II (Qiagen, Hilden, Germany) 10 min at 30 Hz.

The V3-V4 region of the 16S rRNA gene and the ITS2 region were amplified with the 465 primer pairs 343F/806R and ITS3_KYO2/ITS4, respectively, combined with Illumina 466 adapter sequences [66]. PCR amplification was performed as described earlier [66]. A total of 200 ng PCR product from each sample was pooled together and purified through MinElute Gel Extraction Kit (Qiagen, Hilden, Germany). The libraries were sequenced with 2 × 300 bp paired-end reads on MiSeq (Illumina, San Diego, CA, USA) in SB RAS Genomics Core Facility (ICBFM SBRAS, Novosibirsk, Russia). The read data were submitted to the NCBI Short Read Archive under bioproject accession number PRJNA976959.

Raw sequences were analyzed using the UPARSE pipeline [67] with Usearch v11.0.667. The UPARSE pipeline included merging of paired reads, read quality filtering, length trimming, merging of identical reads (dereplication), discarding single reads, removing chimeras and OTU clustering using the UPARSE algorithm. The OTU sequences were assigned a taxonomy using the SINTAX [68] and 16S RDP training set v.18 [69] or for fungi ITS UNITE USEARCH/UTAX v.8.3 as a reference. Alpha diversity metrics were calculated in Usearch.

### 5.7. Data Analyses

Data were checked for Gaussian distribution using the D’Agostino–Pearson omnibus test, and if non-normal, a conservative non-parametric analysis was applied. A nonparametric *t*-test (Mann–Whitney test) was used for comparison of soil populations of the fungus *R. solani*, bacterial (16S) and fungal (ITS) communities. Parametric unpaired *t* tests were used for comparison of rhizoctonia disease on the stems, rhizoctonia disease on tubers, plant biochemical indicators, yield of potatoes, fusarium in the soil and biochemical composition of the tubers. Two-way ANOVA with Sidak post hoc tests were used for comparison of morphometric parameters of plants, fungicidal effects on bacterial strains in vitro, and the number of bacteria and fungi in the dry soil. Data were analyzed using GraphPad Prism v8.0 (GraphPad Software Inc., La Jolla, CA, USA).

## Figures and Tables

**Table 1:** Effect on field season development of *Rhizoctonia solani* development on potato. Treatment: bacteria mix (*B. subtilis* and *B. amyloliquefaciens* 1 × 10^6^ CFU/mL), Control: water.

	Week	Scale of Stem Disease Development (Number per Rating Class)						Damaged Stems (%)	Stem Disease Index (DI)	Biological Effectiveness (on Stems), %
		0	1	2	3	4	5			
Control	4	2.2	0.4	0.2	0.0	0.0	0.0	17.6	4.7	-
	6	0.6	0.4	0.8	0.4	0.6	0.0	78.6	30.0	-
	10	0.2	1.2	0.0	0.6	0	0.6	92.3	40.9	-
Bacteria mix	4	3.8	0.0	0.0	0.0	0.0	0.0	0.0	0.0	100.0
	6	2.8	0.8	0.2	0.0	0.0	0.0	36.8	5.3	86.8
	10	2.4	1.0	0.4	0.2	0.0	0.0	40.0	12.0	70.7

## Data Availability

Data are contained within the article.

## Supplementary Materials

The following supporting information can be downloaded at: https://www.mdpi.com/article/10.3390/jof9121142/s1, Figure S1: Effect of bacteria mix (*Bacillus subtilis* and Bacillus amyloliquefaciens) treatment (1 × 10^6^ CFU) on mass per plant *Solanum tuberosum*; Figure S2: Effect of mix bacteria (*Bacillus subtilis* and *Bacillus amyloliquefaciens*) treatment (1 × 10^6^ CFU) on fungus *Rhizoctonia solani*; Figure S3: Symptoms of development of *Rhizoctonia solani* fungus on tubers of *Solanum tuberosum* after treatment with bacteria mix (*Bacillus subtilis* and *Bacillus amyloliquefaciens*); Figure S4: The number of bacteria (A) and fungi (B) in the dry soil on the 4th week post planting of potatoes *Solanum tuberosum* treated with mix bacteria (*Bacillus subtilis* and *Bacillus amyloliquefaciens*) (1 × 10^6^ CFU) and water (Control); Figure S5: The indicators of the biochemical composition of tubers *Solanum tuberosum*: mass fraction of moisture, the mass fraction of ash in terms of dry matter, crude fiber, crude protein; Figure S6: The indicators of nitrates (A), pH (B), acidity (C) of tubers Solanum tuberosum (nitrates, total acidity, pH) after treatment with bacteria mix (Bacillus subtilis and Bacillus amyloliquefaciens) (1 × 106 CFU) or control (water) treatment.; Figure S7: The experimental design; Table S1: Effect of mix bacteria (*Bacillus subtilis* and *Bacillus amyloliquefaciens*) treatment on the development of rhizoctonia on potato *Solanum tuberosum* during the field season; Table S2: The 16S gene and ITS gene sequences of strains used in the study.
