# Supplementary material for: Bacillus subtilis and Bacillus amyloliquefaciens Mix Suppresses Rhizoctonia Disease and Improves Rhizosphere Microbiome, Growth and Yield of Potato (Solanum tuberosum L.)
Source: J Fungi (Basel). 2023 Nov 25;9(12):1142. doi: 10.3390/jof9121142 (PMC10744094; doi:10.3390/jof9121142)
Supplement: Supplementary file 1 [file jof-09-01142-s001.zip › jof-2689493-supplementary.pdf]

Supplementary information

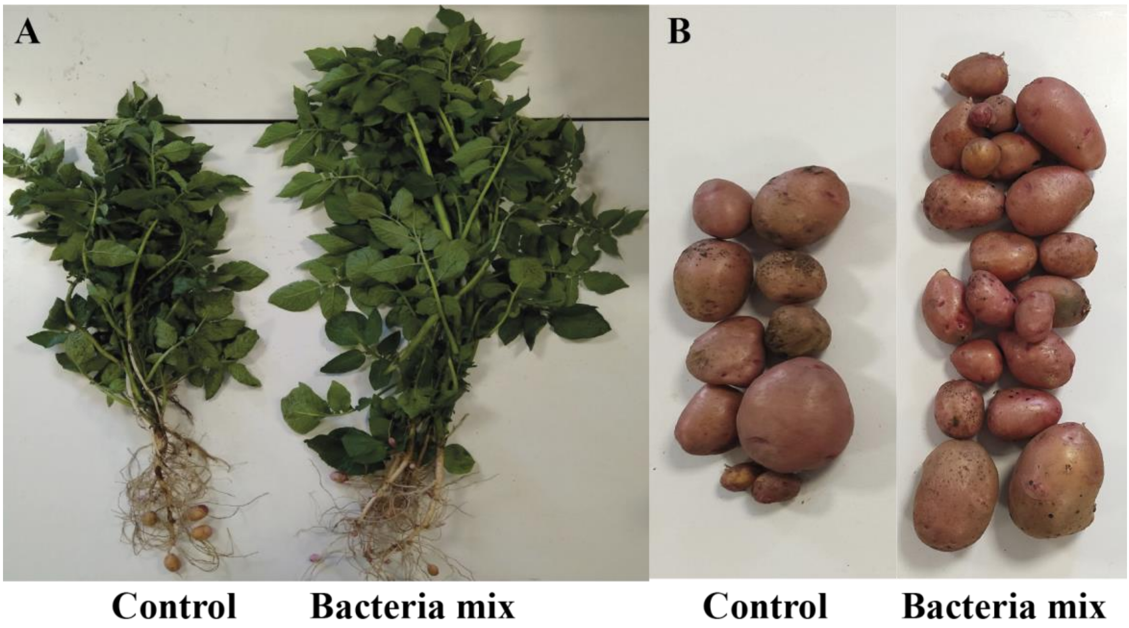

Figure S1. Effect of bacteria mix (*Bacillus subtilis* and Bacillus amyloliquefaciens) treatment (1x10^6 CFU) on mass per plant *Solanum tuberosum* (picture of the potato on the 6th week) (A) and representative yield of potatoes from one plant (B).

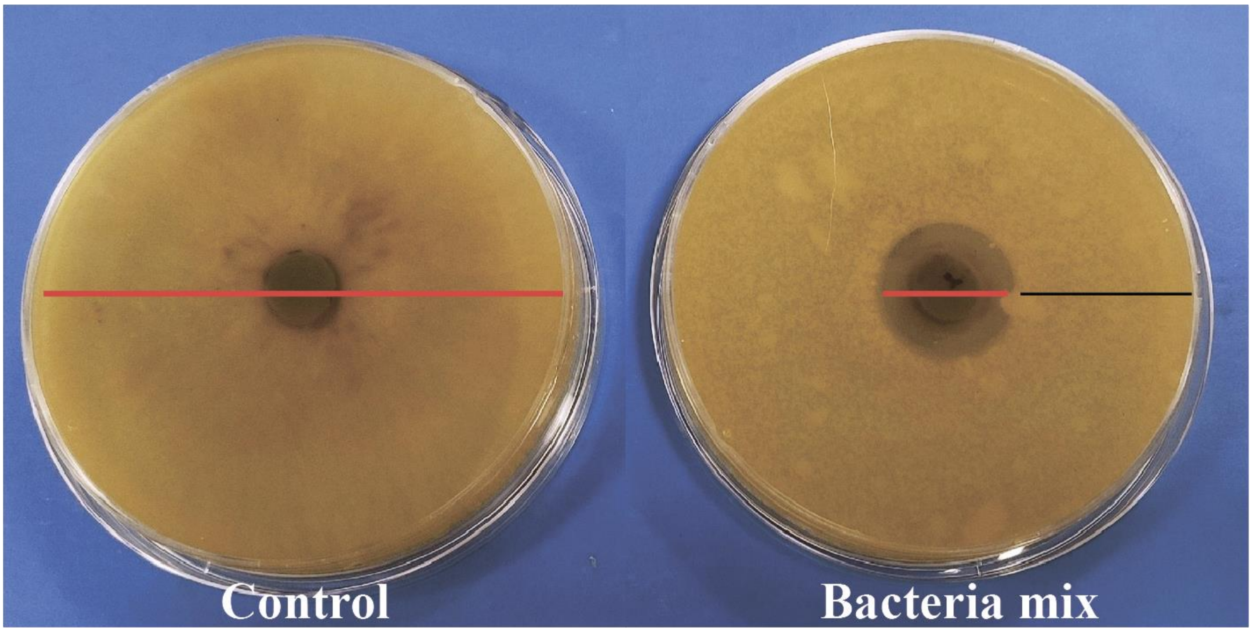

Figure S2. Effect of mix bacteria (*Bacillus subtilis* and *Bacillus amyloliquefaciens*) treatment (1x10^6 CFU) on fungus *Rhizoctonia solani*; Control was treated with water. *R. solani* fungal grow in vitro in presence of the bacteria mix (picture of Petri dishes with control and mix of bacteria (Red lines show diameter of *R. solani* colony, black lines show diameter of bacteria colonies).

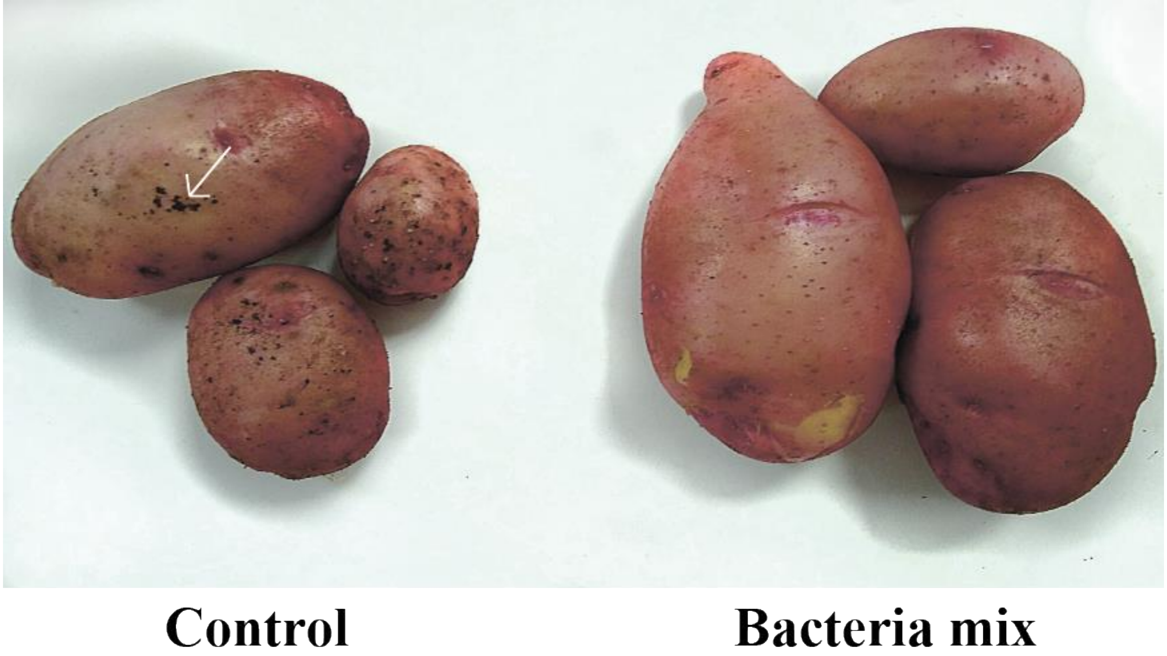

Figure S3. Symptoms of development of *Rhizoctonia solani* fungus on tubers of *Solanum tuberosum* after treatment with bacteria mix (*Bacillus subtilis* and *Bacillus amyloliquefaciens*) ($1x10^6$ CFU) or control (water) treatment. Symptoms are indicated with white arrow.

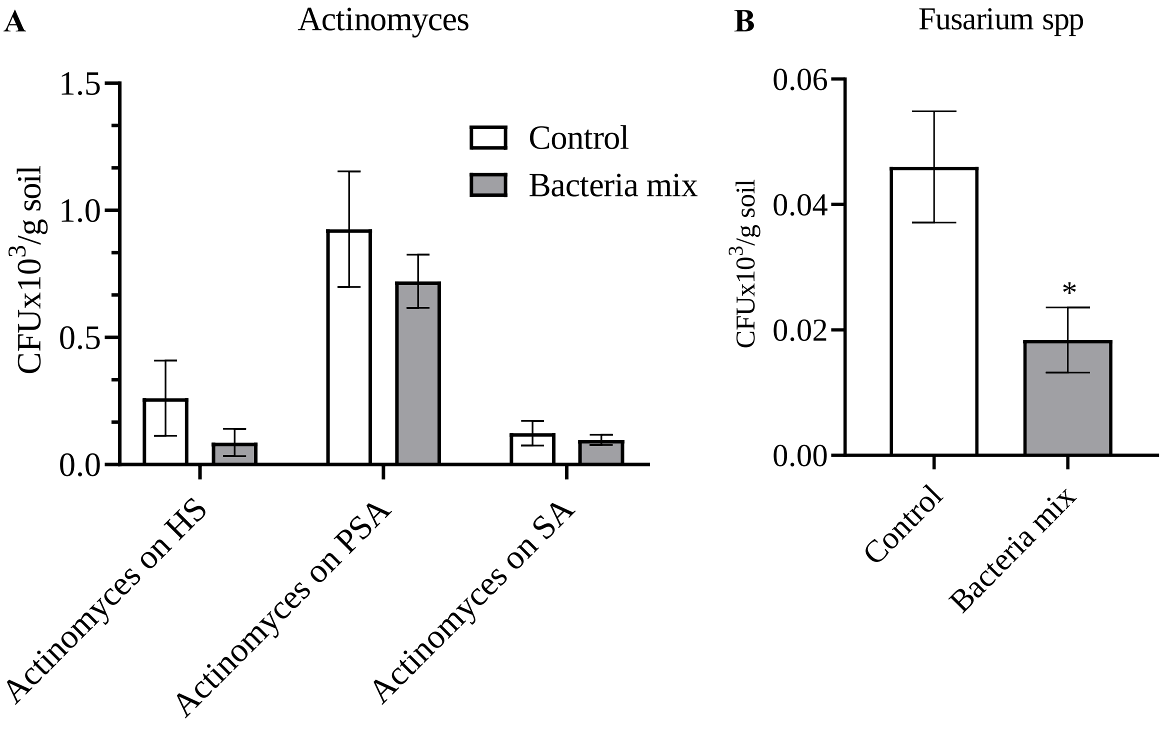

Figure S4. The number of bacteria (A) and fungi (B) in the dry soil on the 4th week post planting of potatoes *Solanum tuberosum* treated with mix bacteria (*Bacillus subtilis* and *Bacillus amyloliquefaciens*) ($1x10^6$ CFU) and water (Control) (* $p \leq 0.05$ compared with control) Microorganisms divided on assimilating mineral nitrogen (PSA media), organic nitrogen (ammonifiers) (MPA media) and cellulosolytic activity (HS media).

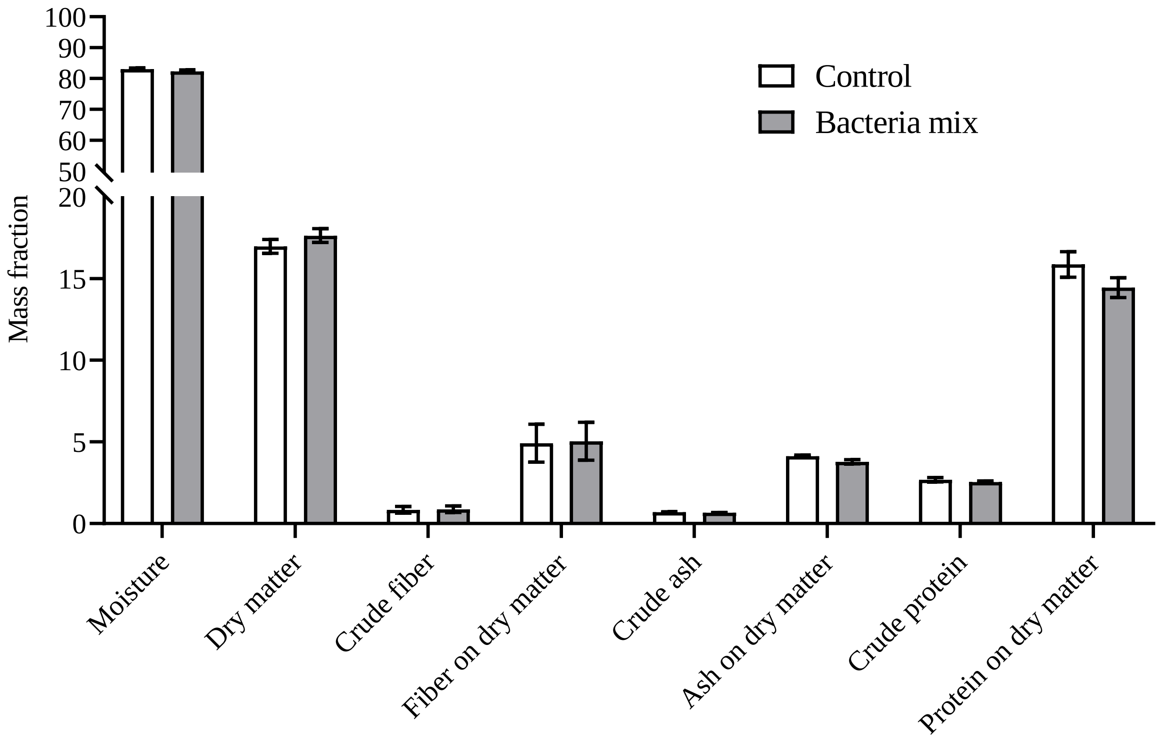

Figure S5. The indicators of the biochemical composition of tubers *Solanum tuberosum* (mass fraction of moisture, the mass fraction of ash in terms of dry matter, crude fibre, crude protein) after treatment with bacteria mix (*Bacillus subtilis* and *Bacillus amyloliquefaciens*) (1x10^6 CFU) or control (water) treatment.

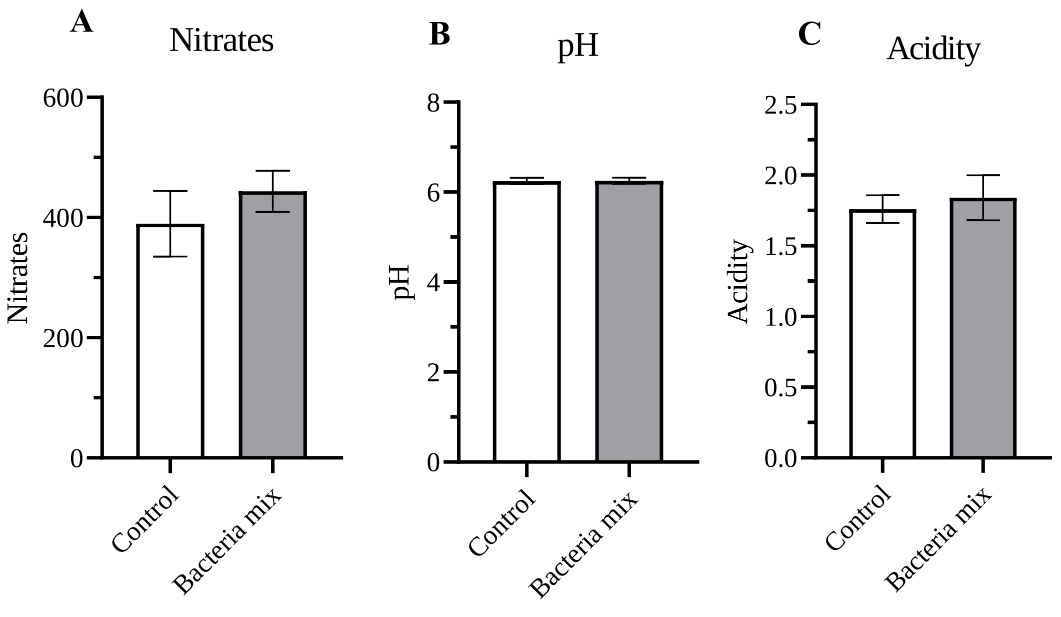

Figure S6. The indicators of nitrates (A), pH (B), acidity (C) of tubers *Solanum tuberosum* (nitrates, total acidity, pH) after treatment with bacteria mix (*Bacillus subtilis* and *Bacillus amyloliquefaciens*) (1x10^6 CFU) or control (water) treatment.

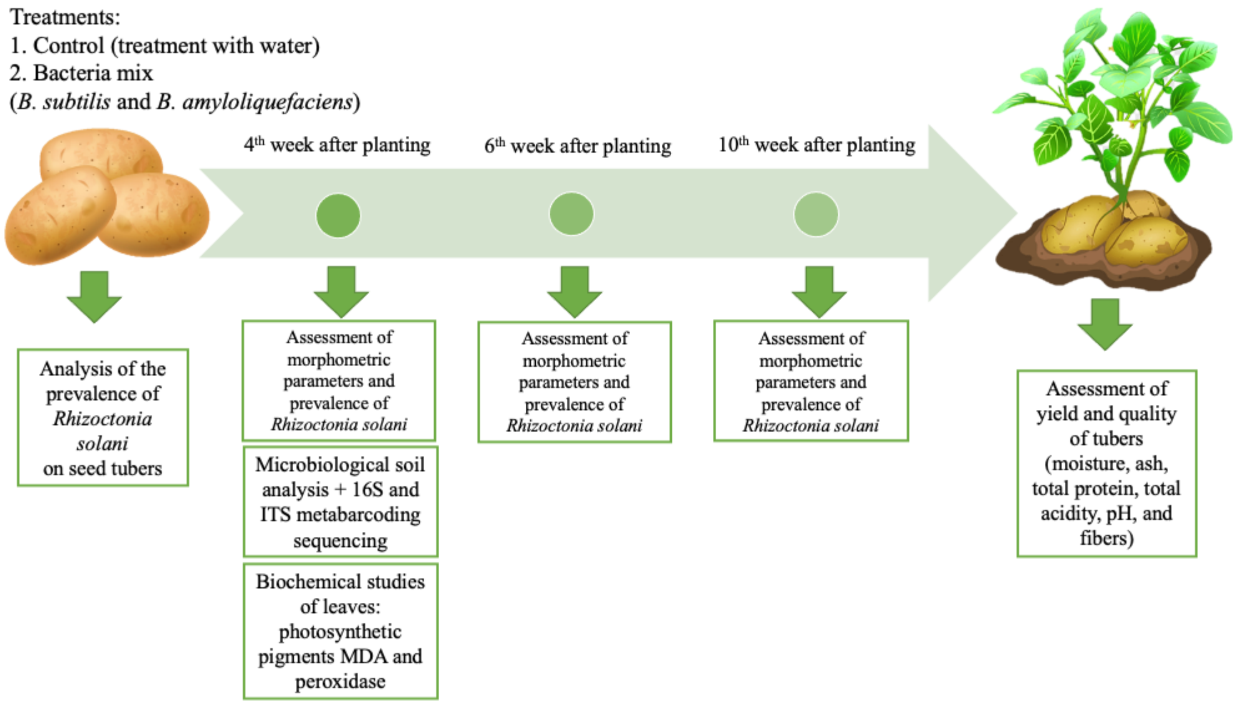

Figure S7. The experimental design

Table S1. Effect of mix bacteria (*Bacillus subtilis* and *Bacillus amyloliquefaciens*) treatment (1x10^6 CFU) on the development of rhizoctonia on potato *Solanum tuberosum* during the field season.

Variant	Accounting date (week)	Damaged stolons (number)		
		total	damaged	fallen
Control	4	0	0	0
	6	5,6	0,8	0
	10	14,6	1,6	0,6
Bacteria mix	4	0	0	0
	6	13,2	0,6	0
	10	29,8	2,6	0,8

Table S2. Sequences of bacterial (16S) and fungal (ITS) genes for species verification.

Species	Collection/ isolation	Gene	Sequence	NCBI analysis
Bacillus amyloliquefaciens	VKPM B-10642	16s	GGATACTTGGACTTACCCTAGCAGCGGCTG GCGCCTCAGGTTACCTCACCGACTTCGGGT GTTACAAACTCTCGTGGTGTGACGGGCGGT GTGTACAAGGCCCGGGAACGTATTCACCGC GGCATGCTGATCCGCGATTACTAGCGATTC CAGCTTCACGCAGTCGAGTTGCAGACTGCG ATCCGAACTGAGAACAGATTTGTGGGATTG GCTTAACCTCGCGGTTTCGCTGCCCTTTGTT CTGTCCATTGTAGCACGTGTGTAGCCCAGG TCATAAGGGGCATGATGATTTGACGTCATC CCCACCTTCCTCCGGTTTGTCACCGGCAGTC ACCTTAGAGTGCCCAACTGAATGCTGGCAA CTAAGATCAAGGGTTGCGCTCGTTGCGGGA	Confirmed

			CTTAACCCAACATCTCACGACACGAGCTGA<br>CGACAACCATGCACCACCTGTCACTCTGCC<br>CCCGAAGGGGACGTCCTATCTCTAGGATTG<br>TCAGAGGATGTCAAGACCTGGTAAGGTTCT<br>TCGCGTTGCTTCGAATTAAACCACATGCTCC<br>ACCGCTTGTGCGGGCCCCCGTCAATTCCTTT<br>GAGTTTCAGTCTTGCGACCGTACTCCCCAG<br>GCGGAGTGCTTAATGCGTTAGCTGCAGCAC<br>TAAGGGGCGGAAACCCCCTAACACTTAGCA<br>CTCATCGTTTACGGCGTGGACTACCAGGGT<br>ATCTAATCCTGTTCGCTCCCCACGCTTTCGC<br>TCCTCAGCGTCAGTTACAGACCAGAGAGTC<br>GCCTTCGCCACTGGTGTTCCTCCACATCTCT<br>ACGCATTTCACCGCTACACGTGGAATTCCA<br>CTCTCCTCTTCTGCACTCAAGTTCCCCAGTT<br>TCCAATGACCCTCCCCGGTTGAGCCGGGGG<br>CTTTCACATCAGACTTAAGAAACCGCCTGC<br>GAGCCCTTTACGCCCAATAAATTCCGGGA<br>CAACGCTTGCCACCCTACCGTATTACCGCG<br>GGCTGCTGGGCACGTAGTTAGCCCGTGCTT<br>TTCTGGTAGGTACCGGTCAAGTGGCCGCCC<br>TATTGGACTGCACGTTGTTCATCCGTAACCA<br>ACCAGAAGCCTTTACGATCCGGAAC	
B. amyloliquefaciens	VKPM B-10643	16s	CATCACTCTAGCAGCGGCTGCGCGCCTGCA<br>AGGTTACCTCACCGACTTCGGGTGTTACAA<br>ACTCTCGTGGTGTGACGGGCGGTGTGTACA<br>AGGCCCGGGAACGTATTCACCGCGGCATGC<br>TGATCCGCGATTACTAGCGATTCCAGCTTCA<br>CGCAGTCGAGTTGCAGACTGCGATCCGAAC<br>TGAGAACAGATTTGTGGGATTGGCTTAACC<br>TCGCGGTTTCGCTGCCCTTTGTTCTGTCCAT<br>TGTAGCACGTGTGTAGCCCAGGTCATAAGG<br>GGCATGATGATTTGACGTCATCCCCACCTTC<br>CTCCGGTTTGTCACCGGCAGTCACCTTAGA<br>GTGCCCAACTGAATGCTGGCAACTAAGATC<br>AAGGGTTGCGCTCGTTGCGGGACTTAACCC<br>AACATCTCACGACACGAGCTGACGACAACC<br>ATGCACCACCTGTCACTCTGCCCCCGAAGG<br>GGACGTCCTATCTCTAGGATTGTCAGAGGA<br>TGTCAAGACCTGGTAAGGTTCTTCGCGTTGC<br>TTCGAATTAAACCACATGCTCCACCGCTTGT<br>GCGGGCCCCCGTCAATTCCTTTGAGTTTCAG<br>TCTTGCGACCGTACTCCCCAGGCGGAGTGC<br>TTAATGCGTTAGCTGCAGCACTAAGGGGCG<br>GAAACCCCCTAACACTTAGCACTCATCGTTT<br>ACGGCGTGGACTACCAGGGTATCTAATCCT<br>GTTCGCTCCCCACGCTTTCGCTCCTCAGCGT<br>CAGTTACAGACCAGAGAGTCGCCTTCGCCA<br>CTGGTGTTCCTCCACATCTCTACGCATTTCA<br>CCGCTACACGTGGAATTCCACTCTCCTCTTC<br>TGCACTCAAGTTCCCCAGTTTCCAATGACCC<br>TCCCCGGGTTGAGCCGGGGGCTTTCACAT<br>CAGACTTAAGAAACCGCCTGCGAGCCCTTT<br>ACGCCCAAATAATTCCCGGACAACGCTCGC<br>CACCTACGTATTACCGGCGGCTGCTGGCAC<br>GGTAGTTAGCCGTGGCTTTTCTGGTTTAG<br>GTACCCGGTCAGTGCCCGCCGTAATTTGGA<br>ACTGCACTTGTTCTTCCCTAACACCAGAGCT<br>TACGATCGGAAAATCGTTCAATCACTCAAG<br>CCGGCGT	Confirmed
B. subtilis	VKPM B-10641	16s	TTACAACTTGACATCGCTCTAGCAGCGGCT<br>GGCGCCTCAGGTTACCTCACCGACTTCGGG<br>TGTTACAAACTCTCGTGGTGTGACGGGCGG	Confirmed

			TGTGTACAAGGCCCGGGAACGTATTCACCGCGGCATGCTGATCCGCGATTACTAGCGATTCCAGCTTCACGCAGTCGAGTTGCAGACTGCGATCCGAACTGAGAACAGATTTGTGGGATTGGCTTAACCTCGCGGTTTCGCTGCCCTTTGTTCTGTCCATTGTAGCACGTGTGTAGCCCAGGTCATAAGGGGCATGATGATTTGACGTCATCCCCACCTTCCTCCGGTTTGTCACCGGCAGTCACCTTAGAGTGCCCAACTGAATGCTGGCAACTAAGATCAAGGGTTGCGCTCGTTGCGGGACTTAACCCAACATCTCACGACACGAGCTGACGACAACCATGCACCACCTGTCACTCTGCCCCCGAAGGGGACGTCCTATCTCTAGGATTGTCAGAGGATGTCAAGACCTGGTAAGGTTCTTCGCGTTGCTTCGAATTAAACCACATGCTCCACCGCTTGTGCGGGCCCCCGTCAATTCCTTTGAGTTTCAGTCTTGCGACCGTACTCCCCAGGCGGAGTGCTTAATGCGTTAGCTGCAGCACTAAGGGGCGGAAACCCCCTAACACTTAGCACTCATCGTTTACGGCGTGGACTACCAGGGTATCTAATCCTGTTCGCTCCCCACGCTTTCGCTCCTCAGCGTCAGTTACAGACCAGAGAGTCGCCTTCGCCACTGGTGTTCCTCCACATCTCTACGCATTTCACCGCTACACGTGGGAATTCCACTCTCCTCTTTCTGCACTCAAGTTCCCCAGTTTCCAATGACCCCTCCCCGGGTTTGAGCCGGGGGGGCTTTTCACATCAAGACTTAAGGAAACCCGCCCTGCGAGGCCCTTTACGCCCGAATAAATTCCCGGACCAACGCTTTGCCCACCCTACCGTATTTACCCGCGGCTGGCTTGGCCACGTAGTTTAGCCCGTTGTCATTTCTTGGATTAAGGTACCCGTCAAAGTGGCCAGACGCGTTAATTAGAACTGGCACGTTGTTCTTCCCGGTAACGACCGAG	
Rhizoctonia solani	Isolated from rhizoctoniosis-affected potato tubers of the Rosara variety (Russia, Novosibirsk)	ITS	GGTAGCAGCATCATAGCGCAACCGAAGCGTGCACTACGAGATCGGTCGCCACGGACGTATGTGACAAGGGGCTATAACACACCCGAAGGTGCCACGTTCCCCCAGCCTTTTTCCATTGGTCAAAATTCGTGCTGGCCCGTAGACTAGGAAATGCACCAAGCAAGAGCAAGGCTGAATCCCAAAGAACGCGACTGACTTCAAGCGTTTCCATTTCAACAATTTCGCGTACTGTTTAACTCTCTCTCCAAAGTGCTTTTCATCTTTCCCTCACGGTACTTGTTCGCTATCGGTCTCTCGCCAATATTTAGCTTTAGATGGAATTTACCACCCATTTTGAGCTGCATTCCCAAACAACTCGACTCGTTGAGAGCACAACACAAAGCACTGGGAGTCCATGTGAAGGACGGGATTCTCACCCTCTATGACGCTCTGTTCCAAGAGACTTGTACATGGTCCAGCACGGATGATGCTTCTCTAGACTACAACTCGGACAGAATACACTGCCAGATTTTAAATTTGAGCTCTTCCCGCTTCACTCGCCGTTACTAGGGGAATCCTTGTTAGTTTCTTTTCCTCCGCTTATTGATATGCTTAAGTTCAGCGGGTAGTCCTACCTGATTTGAGATCAGATCAGAATAATAGTGTCCAAGTCAATGGACTATTAGAAGCGGTTCATCTGCATTTACCTTGGCCACTTTTTACAGTGTCCTCAGCGATAGATAATTTATCACGCTGAGTGGAACCAAGCATAACACTGAGATCCAGCTAATACACAAAGAGGAGCAGGTGTGAAGCTGCAAAAGACCTCCAATACCAAAATCAAACTAATTGAGTTAACAAAAAGATTCATTTTGAAGATTTCATGATACTCAACAGGCAT	Confirmed

			GCTCCAATGGAATACCAAGGAGCGCAAGGT<br>GCGTTCAAAGATTCGATGATTCACTGAATT<br>CTGCAATTCACATTACTTATCGCATTTCGCT<br>GCGTTCTCATCGATGCGAGAGCCAAGAGAT<br>CGGTTTGTTGAACTAAGGTATGGAGATGTG<br>TACATCCATTACATCATTTAAAAATAACTTG<br>GATTTATATGGATGAGTAGACGAGGTCAAT<br>GACTATTA	
